# Inferring the Population Mean with Second-Order Information in Online Social Networks

**DOI:** 10.3390/e20060480

**Published:** 2018-06-20

**Authors:** Saran Chen, Xin Lu, Zhong Liu, Zhongwei Jia

**Affiliations:** 1 College of Systems Engineering, National University of Defense Technology, Changsha 410073, China; 2 School of Business, Central South University, Changsha 410083, China; 3 School of Mathematics and Big Data, Foshan University, Foshan 528000, China; 4 Department of Public Health Sciences, Karolinska Institutet, 17177 Stockholm, Sweden; 5 National Institute of Drug Dependence, Health Science Center, Peking University, Beijing 100191, China

**Keywords:** population mean inference, second-order information, online surveys, sensitive variable

## Abstract

With the increasing use of online social networking platforms, online surveys are widely used in many fields, e.g., public health, business and sociology, to collect samples and to infer the population characteristics through self-reported data of respondents. Although the online surveys can protect the privacy of respondents, self-reporting is challenged by a low response rate and unreliable answers when the survey contains sensitive questions, such as drug use, sexual behaviors, abortion or criminal activity. To overcome this limitation, this paper develops an approach that collects the second-order information of the respondents, i.e., asking them about the characteristics of their friends, instead of asking the respondents’ own characteristics directly. Then, we generate the inference about the population variable with the Hansen-Hurwitz estimator for the two classic sampling strategies (simple random sampling or random walk-based sampling). The method is evaluated by simulations on both artificial and real-world networks. Results show that the method is able to generate population estimates with high accuracy without knowing the respondents’ own characteristics, and the biases of estimates under various settings are relatively small and are within acceptable limits. The new method offers an alternative way for implementing surveys online and is expected to be able to collect more reliable data with improved population inference on sensitive variables.

## 1. Introduction

Online social networking platforms, e.g., Facebook, Twitter, etc., on which users share their daily life and build social relations with others, provide a tremendous amount of data for researchers to study social phenomena and to validate the theoretical models [1,2]. With the increasing use of online social networking platforms, a large number of surveys on different topics have been conducted through online social networks [3], such as political opinions [4,5], sexual behaviors [6,7] and healthcare [8,9]. Compared with the offline surveys such as face-to-face interviews, the online surveys are cost efficient and easy to implement through social networking platforms and can protect the privacy of respondents with the absence of the interviewers [10]. From the samples collected by popular sampling strategies, such as simple random sampling and random walk-based sampling, the population mean is easy to infer when the self-reported data of the respondents’ own characteristics are available [11,12]. When the respondents are randomly selected from the population, the population mean can be estimated by the sample mean [13,14]. When the respondents are selected via a crawler-like random walk, the population mean is typically estimated by a re-weighted correction of the nodal degree [15,16,17]. Although the online surveys can increase the protection of the privacy of respondents, the self-reported data of respondents’ own characteristics are difficult to obtain when the studied topics or characteristics are illegal or sensitive [18], e.g., drug use, sexual behavior, abortion or criminal activity. Previous studies have found that a large proportion of respondents may refuse to participate partly or not at all in online surveys that include sensitive questions and those who attend tend to misreport their own characteristics because the researchers can directly learn their own characteristics from the self-reported data [19,20]. With the low accuracy of the self-reported data, the unbiased estimators of simple random sampling and random walk-based sampling become unreliable.

Several strategies have been developed for surveying of sensitive variables to improve response rate and to reduce misreporting. For example, the randomized response technique (RRT) [21] designs a pair of questions for each respondent in which one question is sensitive and the other is unrelated and innocuous. A randomizing device (e.g., flipping a coin or rolling a die) is used to decide whether a respondent will answer the sensitive question. This procedure is only known to the respondent. Although the interviewers are unaware of which question the respondent is going to answer, the inference can also be conducted on the known probability that the respondent answers the sensitive question. Another method, the item count technique (ICT) [22], is also widely used. This method constructs two lists of items that are identical except for that one is longer by one sensitive item. Then, the respondents are randomly divided into two groups: one group is given the list with the sensitive item, while the other is given the list without the sensitive item. All respondents are asked the number of, but not which, items in the list in which they would participate. Therefore, the estimate of sensitive characteristics can be inferred from the difference between the average number of items in the group without the sensitive item and the number in the group with the sensitive item. Besides RRT and ICT, the three-card method [23] randomly divides respondents into three groups; the respondent of each group is given a question card about whether he or she belongs to a non-sensitive category. Since the three categories in question cards and the sensitive category are all mutually exclusive, the estimate of the sensitive category is one minus the sum of the proportion of the other three categories. The above methods can be easily implemented for online surveys and can be used to conceal respondents’ answers to researchers [20]. However, there are several limitations for these methods. First, although the above strategies indirectly use ways to collect the respondents’ own characteristics, they do not avoid respondents providing their own characteristics, which is the primary reason for introducing the non-response and misreporting biases [18]. Second, the complex design (e.g., the use of randomizing mechanics) may confuse the respondents and make them impatient, so that the respondents may be unable to understand how it works and, hence, may not feel comfortable to trust the study.

**Figure 1:**
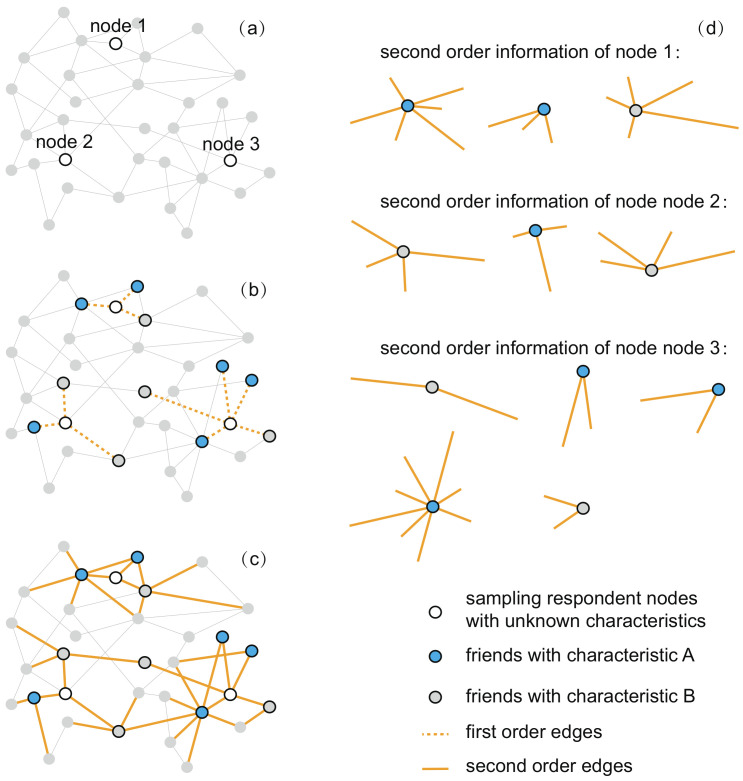
The collection of second-order information of respondent nodes. (**a**) Sampling respondents with unknown characteristics; (**b**) collecting the characteristics of the respondents’ friends; (**c**) collecting the degree (the number of contacts) of these friends; (**d**) the second-order information of respondents.

In this paper, we aim to develop a feasible method that can be applied in the online surveys when the respondents’ own characteristic is difficult to collect (e.g., sensitive characteristics). Inspired by the research showing that people are more willing to talk about their friends’ sensitive characteristics than their own, e.g., drug use [24] and abortion [25], our proposed method is implemented as follows. First, we select a number of respondents according to a sampling strategy. In this paper, two widely-used sampling strategies are considered, i.e., random sampling or random walk-based sampling. Instead of directly asking these respondents’ own information, we collect the second-order information of the respondents in the online social networks. Specifically, we collect the friend lists of these respondents and the number of contacts (degree) of these friends in the social networking platforms and then ask these respondents about the studied variables of their friends in the friend lists, as illustrated in Figure 1. Finally, we develop two Hansen-Hurwitz estimators [26] for generating population estimates with the two sampling strategies.

To verify the effectiveness of the developed method, we implement simulations on both artificial networks and real-world networks. The simulation results show that the bias and variance of the method are small and are within acceptable limits. We believe that, combined with the second-order information, which is easy to obtain in an online social network, this study can provide an easy and feasible approach to infer the population mean on sensitive topics when self-reported data are not available.

## 2. Materials and Methods

### 2.1. Sampling Strategies of Respondents

In this paper, we implement two widely-used sampling strategies to select the respondents from the whole network, i.e., simple random sampling and random walk-based sampling.

Simple random sampling is the most widely-used strategy to create the representative nodes when the sampling frame of the population is available. If the population size is *N*, each node can be randomly selected with equal probability 1/N. If the self-reported data are available and accurate, the population mean can be estimated by the sample mean of simple random sampling [11,17].

Random walk-based sampling is a graph exploration method used in social networks, particularly when the sampling frame of the population is not available. It starts with one or a few seed nodes and selects the next node randomly from the current nodes’ neighbors in the networks. In random walk-based sampling, the inclusion probability of each node is proportional to its degree, so the population mean is typically estimated by the re-weighted correction when the self-reported data are available [17,27].

Although the two classic strategies can provide unbiased estimates on the population mean, they become unavailable when the self-reported data are questionable, such as those for sensitive variables. In the following, we will utilize the respondents selected from the two classic sampling strategies and introduce the new estimators, which can infer the population mean without collecting the respondents’ own characteristics.

### 2.2. Inference with the Second-Order Information

In this paper, when the self-reported data of respondents’ own characteristics are not available, we develop a method to generate the population estimate with the second-order information of respondents, i.e., the characteristics of the respondents’ friends and the degrees of these friends in the online social network.

Specifically, we first select a number of respondents according to a sampling strategy (simple random sampling or random walk-based sampling). Then, we collect the friend lists of these respondents and the degree of these friends in the social network and then ask these respondents about the characteristics of friends in their friend lists. Finally, according to the sampling strategies of the respondents, we estimate the population mean through the provided estimators.

(1) Inference with respondents from simple random sampling:

When the respondents are selected randomly, the inclusion probability of a respondent is 1/N, where *N* is the number of nodes in the network. When a respondent is selected, his/her friends are selected, as well. Therefore, the inclusion probability of a friend node *i* can be calculated by the probability of selecting respondents that connect with this friend:
  (1) $$P_{i}=1-{\left(1-\frac{1}{N}\right)}^{k_{i}},$$
  where $k_{i}$ is the degree of friend node *i*. Then, the proportion of nodes with Characteristic A can be estimated by a Hansen-Hurwitz estimator [26]:
  (2) $$\hat{P}\left(A\right)=\frac{\sum_{i\in A\cap U}\frac{1}{1-{\left(1-\frac{1}{N}\right)}^{k_{i}}}}{\sum_{j\in U}\frac{1}{1-{\left(1-\frac{1}{N}\right)}^{k_{j}}}},$$
  where *U* is the set of all friend nodes and *A* is the set of friend nodes with Characteristic A. We call this estimator SED1.

(2) Inference with respondents from random walk-based sampling:

Similarly, we can also generate the population mean when respondents are selected from the random walk-based sampling. In random walk-based sampling, the inclusion probability of a respondent *s* is proportional to its degree:
  (3) $$P_{s}=\frac{k_{s}}{N<k>},$$
  where <k> is the average degree of the network. Then, the inclusion probability of a friend node *i* can be calculated by the probability of selecting respondents that connect with this friend node:
  (4) $$P_{i}=\sum_{s\in S}\frac{k_{s}}{N<k>},$$
  where *S* is the set of respondents that connect with friend node *i*. The average degree among the friends of a node can be approximated [28,29]:
  (5) $$<k_{n}>=\frac{<k^{2}>}{<k>}.$$
Then, we can use Equation (5) to extrapolate the equation for the inclusion probability of a friend node *i*:
  (6) $$P_{i}=\sum_{s\in S}\frac{k_{s}}{N<k>}\approx \frac{k_{i}<k^{2}>/<k>}{N<k>}\propto k_{i}.$$
Equation (6) indicates that the inclusion probability of a friend node *i* is approximately proportional to its degree. Thus, the proportion of nodes with Characteristic A can be obtained by a Hansen-Hurwitz estimator:
  (7) $$\hat{P}\left(A\right)=\frac{\sum_{i\in A\cap U}\frac{1}{k_{i}}}{\sum_{j\in U}\frac{1}{k_{j}}}.$$
  where *U* is the set of all friend nodes and *A* is the set of friend nodes with Characteristic A. We name this estimator SED2.

### 2.3. Experimental Design

(1) Networks:

We evaluate the developed method on three types of artificial networks and a real-world network: the Erdos–Rényi (ER) network [30], the Barabási–Albert (BA) network [31], the KOSKK (Kumpula-Onnela-Saramäki-Kaski-Kertész) network [32] and the anonymized online MSM (men who have sex with men) social network [33,34]. The artificial networks are configured with *N* = 10,000 nodes and *M* = 100,000 edges. For each type of artificial networks, 10%, 20%, 30% and 40% of randomly selected nodes with Characteristic A is assigned so that the population value for those with Characteristic A, P(A), is 0.1, 0.2, 0.3 and 0.4, respectively. The MSM network has 16,082 nodes and 446,170 edges. Four characteristics are included: age, county (ct), civil status (cs) and profession (pf). The proportions of users with these characteristics are 0.778, 0.388, 0.404 and 0.382, respectively.
The KOSKK model is one of the best models to generate artificial networks which have similar structures to real-life social networks [35], including a skewed degree distribution, small average shortest path lengths, high average clustering coefficient, assortative mixing and community structures. In a KOSKK model, the network to be generated is initialed with *N* nodes and no links. Then, it evolves with three mechanisms: two mechanisms that create links and a mechanism that removes nodes [32].

(i) Local attachment. A node *i* is randomly selected, and one of its neighbors *j* is chosen with probability $w_{ij}/\sum_{j}w_{ij}$, where $w_{ij}$ is the weight on link $e_{ij}$. If *j* has other neighbors besides *i*, one of them (e.g., node *k*) is chosen with probability $w_{jk}/\sum_{k}(w_{jk}-w{}_{ij})$. If no links exist between *i* and *k*, the link is generated with probability $p_{\Delta }$ and set $w_{ki}=w_{0}$. In both cases, the link weight, including $w_{ij}$, $w_{jk}$ and $w_{ki}$, is increased by an amount δ. With larger δ, the generated network will have a stronger community structure.
(ii) Global attachment. Node *i* is connected to a randomly-selected node *l* with probability $p_{r}$ (if no links exist, the probability is one), and the weight in link (i,l) is set to $w_{il}=w_{0}$.
(iii) Node deletion. Any node and its adjacent links (i.e., all of its connections) can be removed with probability $p_{d}$.

When $p_{d}$ is fixed, the average degree is obtained by adjusting $p_{\Delta }$ for each δ. In this paper, we set N=10,000, $w_{0}=1$, $p_{r}=0.0005$, $p_{d}=0.001$, δ=0.6 and the average degree 〈k〉=10. The process runs $10^{8}$ time steps to achieve the stationary state. Due to the node deletion mechanism, a few nodes may be isolated. In that case, we simply connect these nodes randomly to the giant connect component to make all nodes reachable through a path.

(2) Simulation setup:

In each simulation, we first select a number of respondents (10%) through a sampling strategy, i.e., simple random sampling or random walk-based sampling. Then, we collect the characteristics and degrees of the friend nodes of respondents. Finally, we use the collected second-order information to infer the population mean with the SED1 and SED2. For each characteristic, the simulation is repeated 100 times.
In this paper, we use the average of the biases over simulations to measure the performance of the two proposed estimators:
  (8) $$\bar{Bias}=\frac{\sum_{m=1}^{M}\hat{|P_{m}}\left(A\right)-P\left(A\right)|}{M}$$
  where $\hat{P_{m}}\left(A\right)$ is the estimate for the population proportion of nodes with Characteristic A from *m* time simulation, P(A) is the real population proportion and *M* is the number of simulations.

### 2.4. Generation of Networks with Varying Network Parameters

(1) Degree correlation:

In real-world social networks, the nodes do not randomly connect to each other as in the random network model. For example, nodes tend to connect preferentially to other nodes with either similar or opposite degree values [36,37]. In this paper, we use the Pearson correlation coefficient, *r*, to quantify the degree correlation [36]:
  (9) $$r=\frac{M^{-1}\sum_{i}d_{i}k_{i}-\left[M^{-1}\sum_{i}\frac{1}{2}(d_{i}+k_{i}\right){]}^{2}}{M^{-1}\sum_{i}\frac{1}{2}\left(d_{i}^{2}+k_{i}^{2}\right)-{\left[M^{-1}\sum_{i}\frac{1}{2}\left(d_{i}+k_{i}\right)\right]}^{2}},$$
  where *M* is the number of edges in networks and $d_{i}$ and $k_{i}$ are the degree of nodes at the end of the *i*-th edge. The correlation coefficient *r* lies between −1 and one. When r>0, the network shows assortative mixing patterns, i.e., nodes of the network tend to be connected with similar degree values. When r<0, the network is disassortative, i.e., nodes of the network tend to be connected with opposite degree values.
To investigate the effect of degree correlation on the proposed method, we adjust it by a degree-preserving random edge rewiring operation. Specifically, we first randomly pick a pair of edges from the network, $e_{i}=\left(u_{i},v_{i}\right)$ and $e_{j}=\left(u_{j},v_{j}\right)$. Then, we sort these nodes, i.e., $u_{i}$, $v_{i}$, $u_{j}$ and $v_{j}$, by their degree values. If $k_{u_{i}}>k_{u_{j}}>k_{v_{i}}>k_{v_{j}}$, we rewire the two edges as $e_{i}^{{}^{\prime }}=\left(u_{i},u_{j}\right)$ and $e_{j}^{{}^{\prime }}=\left(v_{i},v_{j}\right)$ (as $e_{i}^{{}^{\prime }}=\left(u_{i},v_{j}\right)$ and $e_{j}^{{}^{\prime }}=\left(u_{j},v_{i}\right)$) if we want to increase (decrease) the degree correlation. If new edges do not exist and the recalculated $r^{{}^{\prime }}$ is approaching the desired value, this rewiring operation is kept. Otherwise, the operation is rolled back. The above processes are repeated until $r^{{}^{\prime }}$ reaches the desired value.

(2) Homophily:

Similar to the assortative mixing patterns on degrees, homophily measures the phenomenon that nodes with the same characteristics tend to connect with each other [38,39], which is often observed in real-world social networks. In this paper, we quantify homophily by the probability that nodes connect with neighbors who are similar to themselves with respect to the studied Characteristic A rather than that they connect randomly [34]:
  (10) $$h_{A}=1-S_{AB}/P\left(B\right),$$
  where $S_{AB}$ is the proportion of links originating from nodes with Characteristic A and ending in the same characteristic nodes among all links originating from Characteristic A nodes. When $h_{A}$ = 1, all Characteristic A nodes only connect with Characteristic A nodes themselves, and there is no cross-group connection between Characteristic A nodes and Characteristic B nodes. When $h_{A}$ = 0, there exists no preference of link formation regarding Characteristic A.
To generate the networks with varying homophily, a degree-preserving edge rewiring operation is used in this paper. Let $h_{A}$ be the homophily of the current network and ${h_{A}}^{{}^{\prime }}$ be the desired value. When $h_{A}>{h_{A}}^{{}^{\prime }}$, two with-group edges, $e_{i}=\left(u_{i},v_{i}\right)$ and $e_{j}=\left(u_{j},v_{j}\right)$, are randomly picked (i.e., $u_{i}$, $v_{i}$ are with Characteristic A, and $u_{j}$, $v_{j}$ are with Characteristic B). Then, we rewire the two edges as $e_{i}^{{}^{\prime }}=\left(u_{i},u_{j}\right)$ and $e_{j}^{{}^{\prime }}=\left(v_{i},v_{j}\right)$ to increase the cross-group edges and decrease the homophily. When $h_{A}<{h_{A}}^{{}^{\prime }}$, we pick two cross-group edges randomly and rewire them to form two within-group edges and increase the homophily. The above processes are repeated until $h_{A}$ reaches ${h_{A}}^{{}^{\prime }}$.

(3) Activity ratio:

In real-world networks, the personal networks of nodes are not independent of node characteristics. In this paper, we use the activity ratio (AR) [40] to quantify this characteristic of networks, i.e., the ratio of the mean degree for all nodes with Characteristic A to those with Characteristic B [40]:
  (11) $$AR=\frac{\sum_{i\in A}k_{i}/N_{A}}{\sum_{j\in B}k_{j}/N_{B}},$$
  where $N_{A}$ and $N_{B}$ are the number of nodes with Characteristic A and B, respectively. When AR = 1, the formation of the personal networks is independent of the node characteristics; otherwise, it is affected by the node characteristics.
In this paper, we generate networks with varying activity ratios by a characteristic exchange operation. Let *w* be the activity ratio of the current network and $w^{{}^{\prime }}$ be the value we desire to set. When $w>w^{{}^{\prime }}$, we randomly pick two nodes, node *i* with Characteristic A and node *j* with Characteristic B. The degree of them is $k_{i}$ and $k_{j}$, respectively. If $k_{i}>k_{j}$, we exchange the characteristics of two nodes, i.e., *i* becomes a node with Characteristic B and *j* becomes a node with Characteristic A. If $w<w^{{}^{\prime }}$, we exchange the characteristics of nodes only if $k_{i}<k_{j}$. The above processes are repeated until *w* reaches $w^{{}^{\prime }}$.

## 3. Results

### 3.1. Simulation of Artificial and Real-World Networks

**Figure 2:**
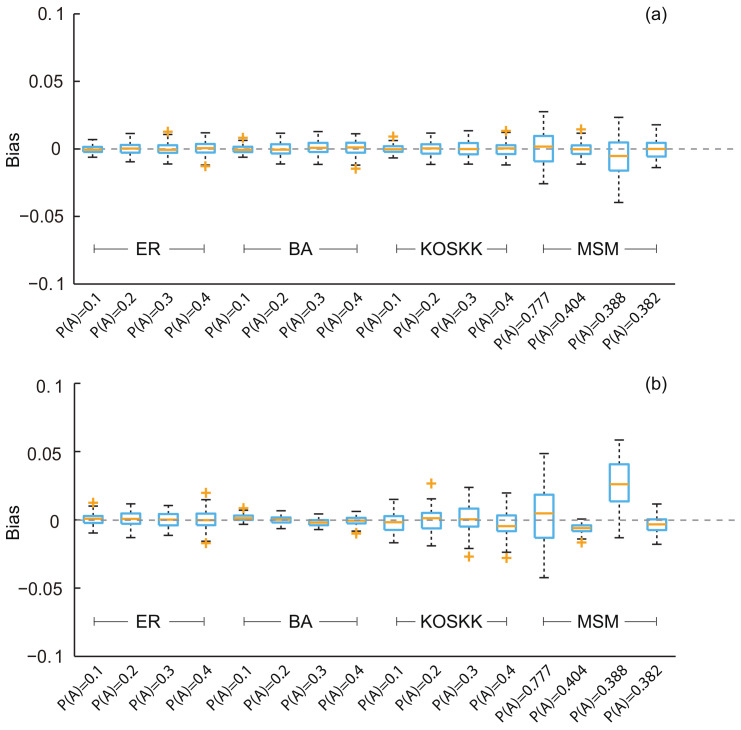
Biases of the population estimates for different networks with varying population properties. (**a**) Respondents were selected by simple random sampling (i.e., estimates obtained from SEC1); (**b**) respondents were selected by random walk-based sampling (i.e., estimates obtained from SEC2). The sampling fraction was 0.1. ER, Erdos–Rényi; BA, Barabási–Albert; KOSKK, Kumpula-Onnela-Saramäki-Kaski-Kertész; MSM, men who have sex with men.

(1) Simulation with simple random sampling:

We first implemented the developed methods on different networks with varying characteristics and studied the performance of the estimator developed for the simple random sampling, i.e., SEC1. The results are shown in Figure 2a. First, we can see that the biases of the estimates are all close to zero. The maximum biases of these characteristics are all below 0.04. Second, the average biases of estimates are all less than 0.005. Among them, the maximum average bias is 0.0045 when P(A) = 0.388 in the MSM network (i.e., characteristic ct). Third, the standard deviations of the biases for the characteristics of the real-world network are larger than those in the artificial networks.
These results indicate that although the performance of the SEC1 for different characteristics is different, the biases and variances are all small. That is to say, the method with the simple random sampling is feasible in both artificial and real-world networks.

(2) Inference with respondents from random walk-based sampling:

Then, we implemented the developed methods on these characteristics and study the performance of the SEC2, which was developed for the random walk-based sampling. Results are shown in Figure 2b. As we can see, these results are similar to those obtained by SEC1. The average biases for the characteristics of artificial networks are all close to zero. The estimates for the characteristics of the real-world networks show slightly larger biases. The biases of these variables deviate from zero: the estimates of age and ct are larger than the real population value (average biases of estimates are 0.003 for age and 0.030 for ct), and the estimates of cs are slightly lower than the real population value (the average bias of estimates is 0.014).
The above results illustrate the effectiveness of the SEC2. Although variation of the performance was observed for different characteristics, the biases of estimates are all small and within acceptable limits (the maximum bias is 0.046 for ct).

### 3.2. Simulation with Varying Network Parameters

Besides the implementation of the developed method in different types of networks with varying population properties, we then focused on the KOSKK network and studied the effects of varying network parameters on the performance of the two estimators. Four network parameters were considered, including average degree, degree correlation, homophily and activity ratio. The baseline network was configured with P(A) = 0.3. In each simulation, 10% of respondents were sampled, and the corresponding second-order information was collected. Then, results were calculated from the two estimators according to the corresponding sampling strategies. All simulations were repeated 100 times.

**Figure 3:**
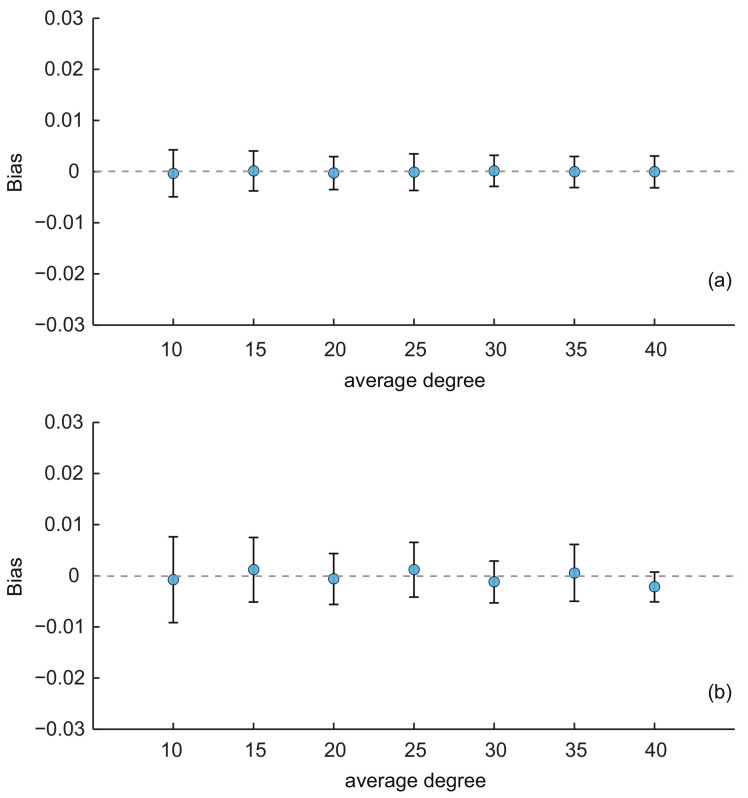
Average biases of the population estimates for the KOSKK networks with varying average degree. (**a**) Respondents were selected by simple random sampling (i.e., estimates obtained from SEC1; (**b**) respondents were selected by random walk-based sampling (i.e., estimates obtained from SEC2). The sampling fraction was 0.1.

(1) Effect of average degree:

First, we investigated the effect of the average network degree on the performance of the two estimators. Besides the baseline network (the average degree was 10), six additional KOSKK networks were generated with the same parameter configurations except that the average degrees were 15, 20, 25, 30, 35 and 40, respectively. Figure 3a shows the results of the SEC1, i.e., when respondents were selected by the simple random sampling. We can see that the average biases are all small and close to zero for varying average degrees. With the increasing of the average degree, the average biases of estimates were almost constant, and their standard deviations slightly decreased. An analysis of variance (ANOVA) test [41] indicated that there was no significant difference of the average biases among estimates with different average degree (*p*-value = 0.94). These results illustrate that the average degree has a limited effect on the performance of SEC1.
Figure 3b shows the results of SEC2, i.e., when respondents are selected by the random walk-based sampling. These results are similar to those in Figure 3a. The average biases of estimates were almost constant, and their standard deviations slightly decreased with the increasing of the average degree. However, an ANOVA test indicated that there existed a significant difference of the average biases among estimates with different average degrees (*p*-value = $1.0\times 10^{-4}$). These results reveal that, although the standard deviation of SEC2 was affected by the average degree, this estimator was also effective when the average degree varied due to the fact that the biases were all close to zero.

**Figure 4:**
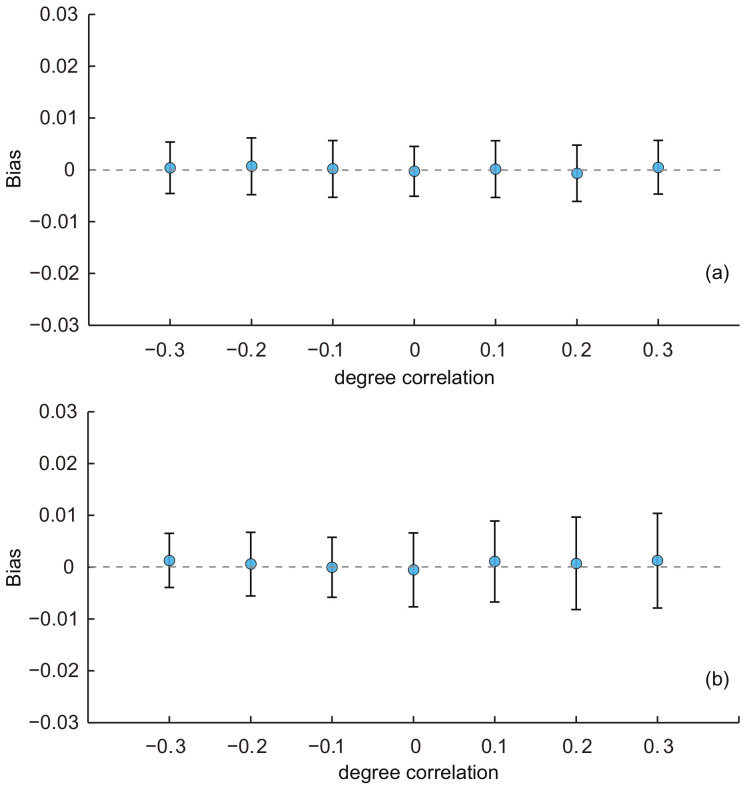
Average biases of the population estimates for the KOSKK networks with varying degree correlations. (**a**) Respondents were selected by simple random sampling (i.e., estimates obtained from SEC1); (**b**) respondents were selected by random walk-based sampling (i.e., estimates obtained from SEC2). The sampling fraction was 0.1.

(2) Effect of degree correlation:

In this part, we used the baseline KOSKK network and adjusted its degree correlation by the rewiring operation to generate seven networks with degree correlations of −0.3, −0.2, 0.1, 0, 0.1, 0.2 and 0.3, respectively. Then, we ran simulations on these networks with the two sampling strategies and compared the biases of estimates to investigate the effect of the degree correlation. Results are shown in Figure 4.
In Figure 4a, we can see that the performance of the SEC1 is almost unaffected by the degree correlation: the average biases and their standard deviations were almost constant with the varying degree correlation. Figure 4b shows the results of the SEC2. We can find that the average biases were almost constant, but the standard deviations of the biases slightly increased with the increasing of the degree correlation. The ANOVA test indicated that there was no significant difference of the average biases among estimates with different degree correlations for both two estimators (*p*-value = 0.58 for SEC1 and 0.51 for SEC2). These results reveal that the degree correlation had a limited effect on the performance of SEC1 and SEC2.

**Figure 5:**
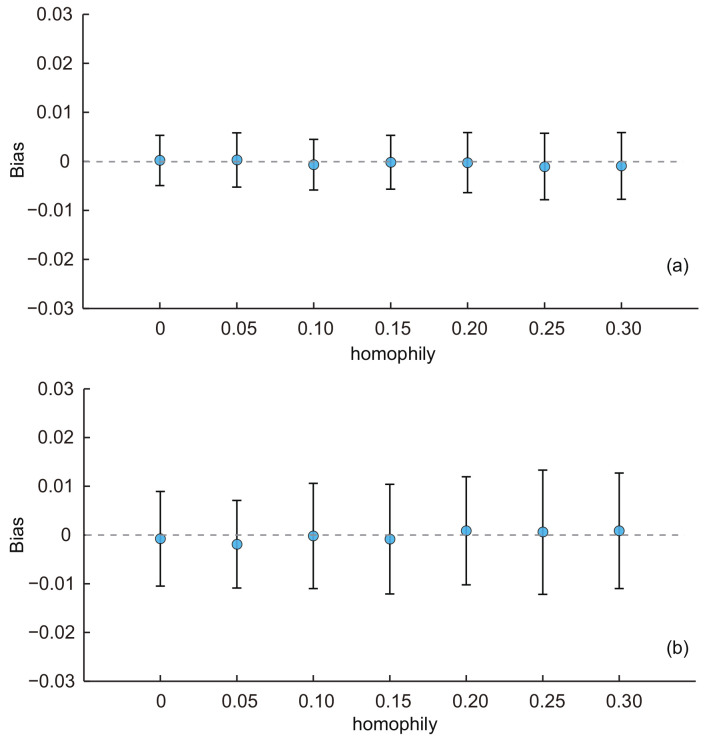
Average biases of the population estimates for the KOSKK networks with varying homophily. (**a**) Respondents were selected by simple random sampling (i.e., estimates obtained from SEC1); (**b**) respondents were selected by random walk-based sampling (i.e., estimates obtained from SEC2). The sampling fraction was 0.1.

(3) Effect of homophily:

Similarly, we used the baseline KOSKK network and adjusted the homophily to generate seven networks with $h_{A}$ of 0, 0.05, 0.10, 0.15, 0.20, 0.25 and 0.3, respectively. The results are shown in Figure 5. From the results, we can see that the average biases of estimates for two estimators were all constant with the increasing of the homophily. The ANOVA test indicated that there was no significant difference of the average biases among estimates with different homophily for both two estimators (*p*-value = 0.56 for SEC1 and 0.54 for SEC2). These results illustrate that the homophily had a limited effect on the performance of the estimators. It is worth noting that traditional estimates for random walk-based sampling are well known for their vulnerability on networks with large homophily. For example, in a study of hidden populations [40], the bias was more than 0.1 when population homophily was high. The unbiasedness of SEC2 implies that the use of second-order information increases the robustness of the population inference and is an indication of potential use for surveys to be implemented with undesirable settings.

**Figure 6:**
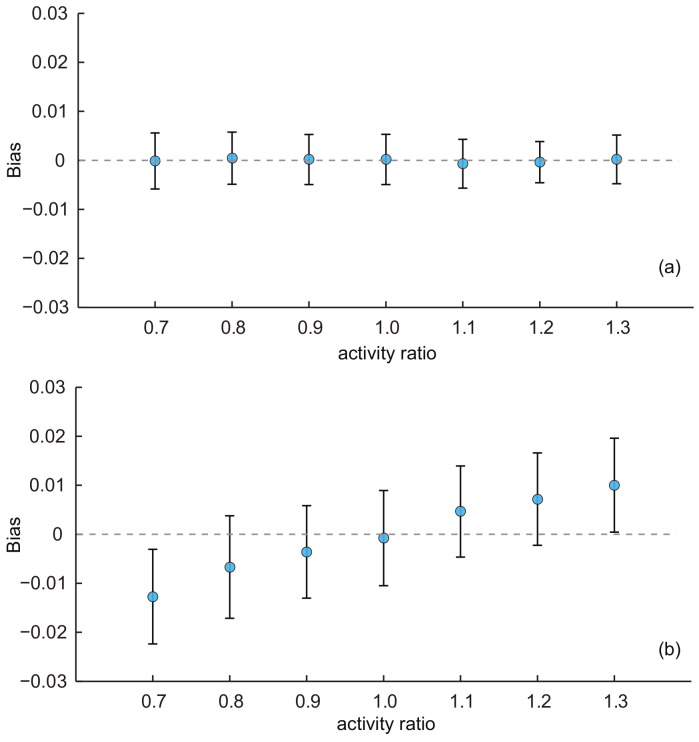
Average biases of the population estimates for the KOSKK networks with varying activity ratios. (**a**) Respondents were selected by simple random sampling (i.e., estimates obtained from SEC1); (**b**) respondents were selected by random walk-based sampling (i.e., estimates obtained from SEC2). The sampling fraction was 0.1.

(4) Effect of activity ratio:

Finally, we used the baseline KOSKK network to generate seven networks with the varying activity ratios of 0.7, 0.8, 0.9, 1.0, 1.1, 1.2 and 1.3 to investigate the effect of the activity ratio on the performance of two proposed estimators.
For SEC1, the average biases were almost constant with varying activity ratios (shown in Figure 6a). The ANOVA test indicated that there was no significant difference of the average biases among estimates with different activity ratios for this estimator (*p*-value = 0.73). For SEC2, we find quite different results in Figure 6b. The performance of SEC2 was greatly affected by the activity ratio. When AR = 1, the average bias is almost zero. However, no matter whether AR is less than or large than one, the average biases deviated from zero: the bias increased with AR, and when AR < 1, the biases tended to be smaller than zero; when AR > 1, the biases tended to be larger than zero. Recalling the results on the MSM real-world network, we also find a similar effect of AR on the estimates: the average biases of characteristic age and ct were larger than zero because their ARs are larger than one (AR = 1.08 for characteristic age and 1.24 for characteristic ct). For characteristic cs (AR = 0.94), the average bias was slightly lower than zero. The most extreme AR existed for the characteristic ct (AR = 1.24) and the average bias of the estimates was the largest: 0.03. On the other hand, for pf (AR = 1.03), which had the AR closest to one, the average bias was the smallest and was almost zero. Note that even SEC2 was affected by the activity ratio, the bias introduced by AR was not large and within acceptable limits (from 0.7–1.3, the biases were all within 0.03).

### 3.3. Simulation with Randomly-Selected Friend Nodes

**Figure 7:**
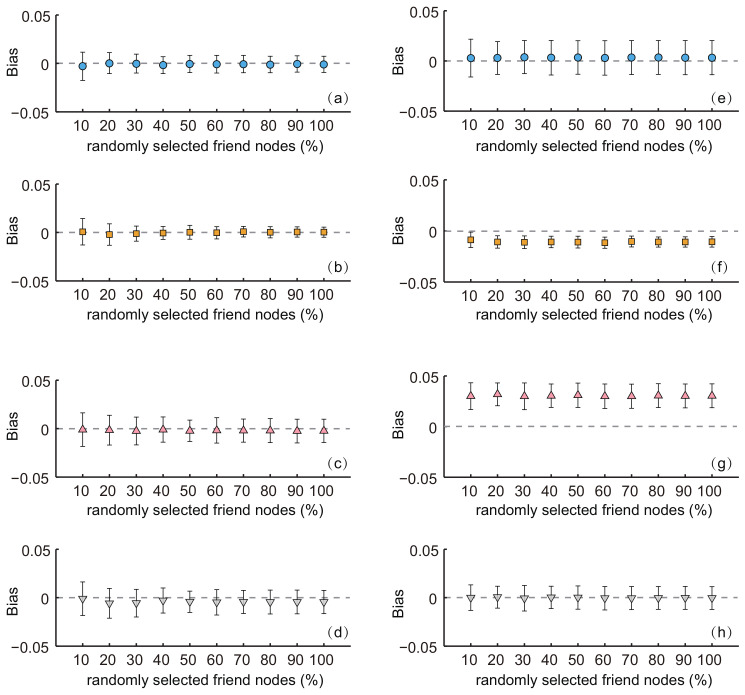
The biases with a varying fraction of randomly-selected friends in the MSM network. (**a**–**d**) Respondents were selected by simple random sampling (i.e., estimates obtained from SEC1). (**e**–**h**) Respondents were selected by random walk-based sampling (i.e., estimates obtained from SEC2). (**a**,**e**) show the results for characteristic age. (**b**,**f**) show results for characteristic civil status (cs). (**c**,**g**) show results for characteristic county (ct). (**d**,**h**) show results for characteristic profession (pf). The sampling fraction was 0.1.

In the online social networks, the number of users’ friends can be very large. For example, some users may have a few hundreds of friends on their friend lists. Collecting the information of such a long list of friends may not be feasible under certain settings (for example, an online crawler program could be prohibited from retrieving large amount of records from the server). To improve the efficiency of our proposed method, it was possible to select a number of friends only randomly from the respondents to produce population estimates. Figure 7 shows the simulation results on the MSM networks.

We can see that, for both estimators, the biases for all characteristics were almost constant with the varying fraction of randomly-selected friends. For each characteristic, the standard deviations of the biases for SEC1 slightly decreased with the increasing of the fraction of randomly-selected friend nodes. The standard deviation for SEC2 was almost constant. The ANOVA tests indicated that there was no significant difference of the average biases among estimates with a different fraction of randomly-selected friend nodes for both estimators (SEC1: *p*-value = 0.98 for age, 0.92 for cs, 0.97 for ct and 0.41 for pf; SEC2: *p*-values for the four characteristics were 0.97, 0.87, 0.98 and 0.99, respectively). That is to say, the estimators were also feasible and effective when a fraction of friends’ information was used.

## 4. Discussions

**Figure 8:**
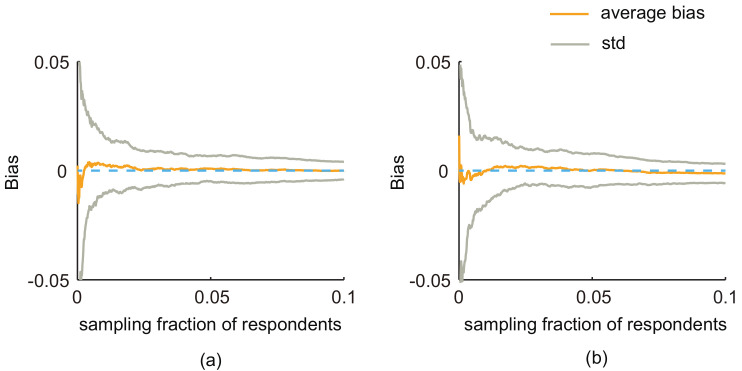
The two estimators can converge to the true population value very quickly. (**a**) Respondents are selected by simple random sampling (i.e., estimates obtained from SEC1); (**b**) respondents are selected by random walk-based sampling (i.e., estimates obtained from SEC2). The results are calculated from the ER network with P(A) = 0.3. The sampling fraction is 0.1.

Although the classic sampling strategies, e.g., simple random sampling and random walk-based sampling, can provide an unbiased estimate of the population variable, their estimators are not available when the self-reported data of respondents’ own characteristics is difficult to obtain in some scenarios, e.g., when the surveys contains sensitive questions. In this paper, we propose an alternative way to infer the population variable when the respondents’ own characteristics were unknown or not reliable in online surveys. Instead of collecting respondents’ own properties, we focused on the second-order information of respondents, which is easy to obtain in online social networks. We develop estimators for two classic sampling strategies, i.e., simple random sampling and random walk-based sampling. The results reveal that the proposed estimators are effective and feasible in both the artificial and real-world networks. Comparing two estimators, the variance of SEC1 are always smaller than that of SEC2 in the same settings. Although the performance of the estimator depended on the random walk-based sampling, i.e., SEC2, is affected by the activity ratio; the bias is small and is within acceptable limits. The advantages of the developed method include: First, using the second-order information avoids the collection of the respondents’ own characteristic, which is the main reason introducing the non-response and misreporting biases when the desired variables are sensitive. Second, the proposed method can be easily implemented with existing sampling strategies; the simulation results show that the proposed estimators can provide estimates with small biases. Third, the estimates quickly converge to the true population value. As shown in Figure 8, when the sampling fraction of respondents reaches 0.02, the biases are all below 0.001 in the ER network with P(A) = 0.3 (we also obtain similar results in other types of artificial networks). That is to say, the proposed method is able to produce reliable estimates with small samples of respondents. Fourth, the second-order information is easy to collect without any additional processes or supporting props embedded in the surveys.

There are some limitations in our study. First, although the bias and variance of SEC1 are very small, the random selection of respondents may be hard to implement in real implementation on the online social networks. One way to solve this problem is to randomly generate users’ ID number and select the corresponding users when the coding rule of the ID number is available (i.e., when there is a sampling frame). Second, the respondents may be more confident to inform about just a few close friends, collecting the information of all nodes in their friend lists may be difficult. Fortunately, we have seen that the estimates with only a fraction of friends’ information being used also have high accuracy. Lastly, empirical studies are to be implemented to evaluate the effectiveness and feasibility of the proposed method in the future.

## 5. Conclusions

In conclusion, the results well illustrate that the proposed method provides a feasible and effective way to infer the population variables on the online surveys when the respondents’ own characteristics are not available. In this paper, we only focus on two classic sampling strategies of respondents, i.e., simple random sampling and random walk-based sampling. We believe that using the framework of the method and the second-order information, other estimators can be constructed through the inclusion probability of friend nodes according to a certain sampling strategy. Besides, the proposed estimators have the potential to be used in many other settings, e.g., monitoring online public opinions and inferring the missing data. Further empirical study of the method is needed, especially to test its performance with the traditional strategies, i.e., RRT, on the online social networks.
